# FDG-PET/CT pitfalls in gynecological and genitourinary oncological imaging

**DOI:** 10.1186/1470-7330-15-S1-P42

**Published:** 2015-10-02

**Authors:** A Lakhani, S Khan, N Bharwani, V Stewart, A Rockall, T Barwick, S Khan

**Affiliations:** 1 Imperial College Healthcare NHS Trust, London, UK

## Learning objectives

1. To understand the role of FDG PET/CT imaging in the multimodality investigation of gynecological and genitourinary cancers.

2. To describe the mechanism of action and technical pitfalls of FDG-PET/CT.

3. To highlight key imaging features of physiological and non-physiological FDG uptake and show how this is essential for interpretation of gynecological and genitourinary FDG-PET/CT studies.

4. to review the pathophysiological mechanisms leading to potentially false-positive and false-negative assessments.

## Content organisation

Introduction of FDG-PET/CT

∘ Mechanism of action

∘ Role in gynecological and genitourinary oncological imaging

∘ FDG-PET/CT imaging protocols

False positives in gynecological and genitourinary oncological imaging:

∘ Physiological FDG-PET uptake – pictorial examples of uptake in endometrium and ovaries

∘ Non-physiological FDG-PET uptake – pictorial examples of pelvic inflammatory disease, fibroids, endometriosis

False negatives in gynecological and genitourinary oncological imaging:

∘ Physiological FDG-PET uptake – pictorial examples of urinary excretion masking malignant lesions

∘ No/low FDG uptake – pictorial examples of necrotic lymphadenopathy and low grade tumours

∘ Artefacts

Pearls explaining how to minimise false interpretation

## Conclusion

FDG-PET/CT has a useful role in gynecological and genitourinary oncological imaging. However, understanding of physiological and non-physiological FDG- PET uptake is vital to understand potential false positive and false negatives in interpretation.

FDG PET/CT should be used as one part of the multimodality investigation of gynecological and genitourinary cancers.

